# Efficacy of Artificial Intelligence-Assisted Psychotherapy in Patients With Anxiety Disorders: A Prospective, National Multicenter Randomized Controlled Trial Protocol

**DOI:** 10.3389/fpsyt.2021.799917

**Published:** 2022-01-20

**Authors:** Shanshan Su, Yuan Wang, Wenhui Jiang, Wenqing Zhao, Rui Gao, Yanru Wu, Jing Tao, Yousong Su, Jie Zhang, Kangzheng Li, Zhuojun Zhang, Min Zhao, Zhen Wang, Yanli Luo, Xiao Huang, Lanlan Wang, Xiaoping Wang, Yi Li, Qiufang Jia, Lianzi Wang, Huafang Li, Jingjing Huang, Jianyin Qiu, Yifeng Xu

**Affiliations:** ^1^Shanghai Mental Health Center, Shanghai Jiao Tong University School of Medicine, Shanghai, China; ^2^ChuanYu (Shanghai) Education Technology Co., Ltd., Shanghai, China; ^3^Renji Hospital, School of Medicine, Shanghai Jiao Tong University, Shanghai, China; ^4^Zhongshan Hospital, Fudan University, Shanghai, China; ^5^Shanghai General Hospital, School of Medicine, Shanghai Jiao Tong University, Shanghai, China; ^6^Department of Psychiatry, The Second Xiangya Hospital of Central South University, Changsha, China; ^7^Department of Psychiatry, Wuhan Mental Health Center, Wuhan, China; ^8^Department of Psychiatry, Suzhou Guangji Hospital, Suzhou, China; ^9^Department of Clinical Psychology, The Fourth People's Hospital of Wuhu, Wuhu, China

**Keywords:** anxiety disorder, artificial intelligence, psychotherapy, randomized controlled single-blind trial, protocol

## Abstract

**Background:**

Anxiety disorders have the highest prevalence of all psychiatric disorders in China. Medication and psychotherapy are two main treatment approaches for this group of disorders, and when used in combinations are significantly more beneficial than medication alone. The resources are insufficient. The availability of psychotherapy is low due to the limited resources. Artificial intelligence (AI)-assisted psychotherapy offers an opportunity to develop an efficient and standardized psychotherapy model and improve the availability of psychotherapy, which is key to improve the clinical efficacy of anxiety disorder treatments.

**Objectives:**

The present protocol aims to determine whether medication plus AI-assisted psychotherapy has greater efficacy than medication alone in the treatment of anxiety disorders.

**Methods:**

We will recruit patients in eight hospitals in China. Seven hundred and eight patients with anxiety disorders will be randomly allocated on a 1:1 basis to either medication plus AI-assisted psychotherapy group, or medication alone group. We have built an AI psychotherapy robot named XIAO AN. In this study we will deliver psychotherapy to patients in the medication plus AI-assisted psychotherapy group. Patients will be assessed at baseline and at the end of week 2, 4, 8, and 12. Follow-up assessments will be conducted at 3 and 6 months posttreatment. The primary outcome is change of Hamilton Anxiety Rating Scale (HAMA) score from baseline the end of 12-week treatment. A secondary efficacy outcome will be improvement in treatment at an early stage (score reduction in HAMA ≥25% after 2 weeks of treatment). Other measurements include Hamilton Depression Scale, Clinical Global Impression, Treatment Emergent Symptom Scale, Social Disability Screening Schedule, Insomnia Severity Index and so on. Scales will be assessed by independent raters who are blind to treatment allocation and analyses will be conducted by a statistician who is also blind to treatment allocation.

**Discussion:**

This will be the first multicentered randomized controlled single-blind trial in China to assess the efficacy of medication plus AI-assisted psychotherapy compared with medication alone for anxiety disorders. The study has the potential to address the limitations of the limited availability of psychotherapy, and to augment the efficacy of the treatment of anxiety disorders in China.

## Introduction

### Background

Anxiety disorders are a cluster of psychiatric disorders that are characterized by symptoms of anxiety. They are the most prevalent class of psychiatric disorders worldwide. The average lifetime prevalence of any anxiety disorder is ~16% and the average 12-month prevalence is ~11% (1). Generally speaking, the prevalence varies widely across different studies, in which higher prevalence is estimated in Western developed countries than in developing countries (1). In the recent national epidemiological study in China, anxiety disorders were the most prevalent mental disorders, with a lifetime prevalence of 7.6% and an annual prevalence of 5.0% (2). The disease burden of these disorders will grow significantly.

In clinical practice, psychotherapy can be used for the treatment of anxiety disorders alone or to optimize the effect of medical treatment with evidence-based studies showing that psychotherapy combined with medication is significantly more beneficial than medication alone (3). Guidelines for anxiety disorders in different countries recommend psychotherapy as the first-line treatment, such as cognitive behavioral therapy, relaxation therapy, systematic desensitization, exposure-response prevention, and other psychotherapy (4–6). However, providing any of these interventions requires professional and experienced therapists, but with limited numbers of qualified therapists, especially in underdeveloped or isolated areas, many patients don't have the opportunity to receive standardized and appropriate treatments. In fact, only 27% of the patients who have received psychotherapy actually got standardized psychotherapy (7).

In response to the lack of availability of standardized face-to-face psychotherapy, artificial Intelligence (AI)-assisted psychotherapy offers an alternative approach. Multiple meta-analyses have shown that Internet Cognitive Behavior Therapy (iCBT) and Computerized Cognitive Behavior Therapy (cCBT) have a significant treatment effect on anxiety disorders and other disorders (8–10). For example, *Woebot*, a fully automated conversational agent can assist patients to identify emotions and acquire skills to reduce anxiety levels, and to reduce symptoms (11). Our team is one of the earliest teams in the field of exploring AI-assisted psychological treatment for anxiety disorders in China. In the early stage, we have developed the human-computer interaction mode of online micro-video therapy to improve the mental resilience of patients with obvious anxiety and depression, and we have developed computerized cognitive behavioral therapy for people with obsessive-compulsive disorder and substance-use disorders (12–14).

To sum up, in order to ameliorate the current situation of the lack of treatment of anxiety disorders, it is necessary to find optimized, efficient and standardized treatment technology. AI-assisted psychotherapy has the potential to do this. We have developed XIAO AN as an AI-assisted psychotherapy robot. XIAO AN is designed to monitor mood and deliver psychotherapy mainly based on Cognitive Behavior Therapy (CBT) in a brief and integrated format, not to replace therapist. It is now necessary to test whether this approach will be beneficial for patients. This proposed study will be the first multicentered randomized controlled trial in China to assess the efficacy of medication plus AI-assisted psychotherapy for anxiety disorders compared with medication alone.

### Objectives

A national multi-center prospective, randomized controlled clinical trial was designed to establish the efficacy of AI-assisted psychotherapy for anxiety disorders in 8 research centers across China.

Specifically, the objective of this trial is to determine whether medication plus AI-assisted psychotherapy has greater efficacy than medication alone in the treatment of anxiety disorders.

## Methods

### Ethics and Informed Consent

This study was approved by Ethics Committee of Shanghai Mental Health Center (SMHC) as the main center and also Ethics Committees of other seven hospitals (Renji Hospital, Zhongshan Hospital, Shanghai General Hospital, Suzhou Guangji Hospital, Wuhan Mental Health Center, the Second Xiangya Hospital of Central South University, and the Fourth People's Hospital of Wuhu) as the subcenters. Written informed consent will be obtained before a screening assessment by trained researchers. Patients will be informed that they will volunteer to participant this study and they have the right to withdraw from the study at any time. If they withdraw, it will bring none negative consequences to them. During all the process of the trial, possible adverse reactions will be monitored. We will do our best to protect the privacy and safety of the patients. The trial was designed and will be conducted based on the principles of good clinical practice and reported according to the CONSORT statement (15, 16). This study has been registered on ClinicalTrials.gov (NCT04515173).

### Setting and Recruitment

**Figure 1:**
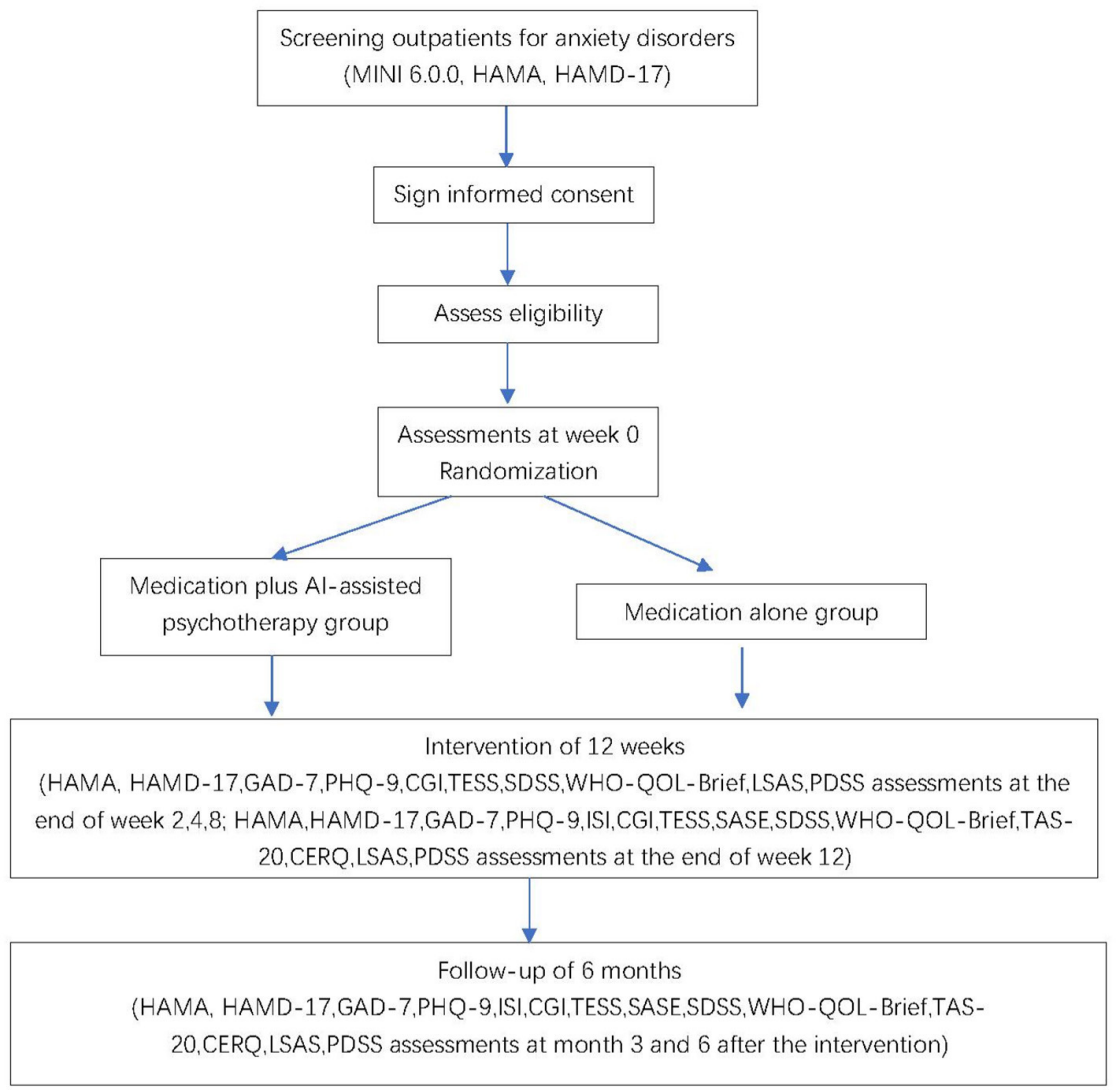
Study design and measurement time point.

We will recruit patients in eight hospitals in China. These sites include Shanghai Mental Health Center, Renji Hospital, Zhongshan Hospital, Shanghai General Hospital, Suzhou Guangji Hospital, Wuhan Mental Health Center, the Second Xiangya Hospital of Central South University, and the Fourth People's Hospital of Wuhu. We will recruit patients with anxiety disorders, including generalized anxiety disorder, panic disorder, agoraphobia, social anxiety disorder, and specific phobia. New outpatients at each of these eight sites who are diagnosed with an anxiety disorder will get a pre-screening individually for about 15 mins by a trained researcher. Pre-screening will introduce the study and invite patients to participate. Those patients who are willing to participate will firstly sign written informed consent forms, then proceed into a screening visit. In the screening visit, they will be assessed to confirm the diagnosis of a DSM-5 anxiety disorder using the Mini International Neuro-psychiatry Interview (MINI 6.0.0) and other eligibility criteria. All the assessments will be administered by qualified staff at each center. All the raters have been trained to high levels of inter-rater reliability (kappa = 0.85). Training of raters for the MINI and other scales includes studying an expert administration firstly and then assessment of patients under the supervision of a qualified trainer. Patients who meet inclusion criteria (show later in section Participants and Eligibility) will proceed into the next step. The trial flow chart is shown in Figure 1.

### Trial Design

This study is a multicenter, parallel, superiority, two-arm, randomized controlled single-blind trial with participants recruited from eight hospitals. Seven hundred and eight outpatients will be randomly assigned on a 1:1 basis to each of the treatment conditions. There will be 354 participants assigned to each group. We consider the medication plus AI-assisted psychotherapy treatment group as the testing group while the medication alone treatment group as the control group.

### Participants and Eligibility

Patients who are eligible need to (a) be between the ages of 18–60 years, (b) meet DSM-5 criteria for an anxiety disorder, including generalized anxiety disorder, panic disorder, agoraphobia, social anxiety disorder, and specific phobia, (c) score>14 on the Hamilton Anxiety Rating Scale (HAMA), (d) be psychotropic medication naïve or have discontinued medication for at least 8 weeks prior to the screening visit, (e) be able to use a computer and smart phone skillfully, and (f) provide written informed consent.

Candidates will be excluded if they: (a) have a severe concurrent medical condition, (b) are at high self-injury or suicide risk, (c) are suffering from a serious physical disease, neurological disease, or substance abuse, (d) have a lifetime history of other DSM-5 mental disorders, (e) are experiencing major life changes, (f) have received systematic drug therapy, psychological therapy or modified electric convulsive therapy (MECT) treatment within 3 months before enrollment, (g) are pregnant or in the lactation period.

### Randomization and Blinding

In this study, a multi-center randomized controlled single-blind design has been adopted. Each site is being used as a stratified factor for stratified block randomization, and each site competes to enroll the subjects. We use the sealed envelope randomization method. Once a patient is deemed eligible to participate, researchers at the relevant site will open the particular numbered envelope to allocate the patient to his or her study condition, either medication plus AI-assisted psychotherapy group or medication alone group. As blinding to patients and researchers is not possible in psychotherapy trials, this trial will be single-blind. The researcher who does randomization is in charge of contacting participants and arranging independent rater to conduct certain assessment at each visit. The assessment time arranging and content in both groups are same. All the measurement will be assessed by independent raters who are unaware of the treatment allocation of patients.

Allocation of patients will be recorded in a separate assessment log which cannot be accessed by raters. In order to ascertain the blinding, patients will be informed not to reveal their treatment during the blinding assessments, and the raters will be asked to document whether patients told them the allocation. The statisticians will also be blind to group allocation.

### Intervention

#### Medication

Patients in both treatment groups will be prescribed anxiolytics. Anxiolytics include selective serotonin reuptake inhibitors (SSRIs), Selective 5-HT and NE reuptake inhibitors (SNRIs), buspirone, tandospirone, and so on, which are approved by the Chinese Food and Drug Administration (CFDA) for the treatment of anxiety disorders. The type and dose of anxiolytics will be based on clinician recommendation according to usual practice. The dosage could be titrated and adjusted as needed, with the maximum dose not exceeding dosage guidelines. Patients with sleep problems will be prescribed benzodiazepines or non-benzodiazepines as sleeping drugs if needed, but not for more than 2 weeks. Other psychotropic drugs are not allowed to use. All the patients should follow the prescription at least 12 weeks of acute treatment and will be asked to keep a daily record of medication use. Non-adherence should be recorded if there is any. Medication use will be documented during the scheduled assessments.

#### AI-Assisted Psychotherapy

##### Technical Overview and Customization

Patients assigned to the medication plus AI-assisted psychotherapy group will receive 12 weekly sessions of AI-assisted psychotherapy. The therapist is XIAO AN. XIAO AN is the name of the virtual psychotherapist designed by Shanghai Mental Health Center (SMHC). The Chinese character for “AN” means quiet, peaceful and safe, which symbolizes a state of mind free from anxiety.

XIAO AN is an AI-based psychological chatbot app which is designed to address the current situation of lack of psychotherapy resources, but not to replace the role of therapists. The knowledge structure and content of XIAO AN were selected, discussed and developed by psychiatric and psychological professionals at SMHC. User experience testing was conducted among a group of medical students and hospital staff, including medical-related staff and non-medical-related staff. We have been continually optimizing the structure and content of XIAO AN over the last year.

The app is installed on an Android tablet. Patients are treated by XIAO AN in a particular consulting room for this study in the relevant hospital. The researchers monitor the patients' treatment via video, and as XIAO AN does not have the crisis management function, if there is a risk of self-injury or suicide, the researchers will recommend patients to pause the process and refer the patient to emergency medical treatment.

##### Integrative-Support-Approach

The psychotherapy composite modules for each patient will be informed by the results of the psychological assessment. The treatment sessions last 15–25 mins and are delivered once a week, for 12 sessions in total.

XIAO AN and the patient interact “face to face,” with XIAO AN delivering therapy that is mainly based on CBT combining psychoeducation, mindfulness-based therapy and interpersonal psychotherapy. For the purpose of relieving anxiety, the therapy embodies regulation of emotion, cognition, behavior, and interpersonal functions. At the end of each session, self-help homework or tasks are assigned, and there is a brief review of the session content, so that patients have the opportunity to apply learnt techniques to their daily life in order to expand and consolidate the effects of the therapy.

In total, there are 15 treatment modules: one episode of psychoeducation about anxiety disorders, two episodes of mindfulness therapy, two episodes of cognitive reconstruction, two episodes of relaxation training, one episode of problem solving, one episode of accumulation of positive emotions, one episode of STOP technology, one episode of self-soothing, one episode of interpersonal efficacy, two episodes of insomnia coping, and one episode of exposure therapy. For each patient, 12 specific treatment modules are recommended according to his or her pattern of anxiety symptoms and sleep problems. For example, treatment modules for patients with agoraphobia and social anxiety disorder will contain an exposure treatment module, while patients having significant sleep difficulties will complete a insomnia coping module.

XIAO AN is designed to reply in an empathic and supportive way in response to the patients' information. For example, if patients rated their anxiety level as more than 8 (ranged 0–10, a higher score means more severe symptom), XIAO AN will respond “I know you are having a hard time, maybe hoping someone or something can help you stop the suffering. Now I'm here, staying with you.” If patients completed an exposure training module, XIAO AN will say “You are doing great. You are so brave. It's very encouraging.”

### Participant Timeline

Each participant will complete the psychological assessment including symptom measurement at baseline and at the end of weeks 2, 4, 8, and 12. Follow-up assessments will be conducted at 3 and 6 months after the 12-week treatment (Table 1).

**Table 1:** Study assessment tools and assessment time schedule.

**Timepoint**	**Enrollment**	**Randomization**	**Postrandomization**					
	**–t1**	**t0**	**t1**	**t2**	**t3**	**t4**	**t5**	**t6**
		**0 weeks**	**2 weeks**	**4 weeks**	**8 weeks**	**12 weeks**	**3 months**	**6 months**
MINI6.0.0	■							
Demographic information	■							
HAMA	■	■	■	■	■	■	■	■
HAMD-17	■	■	■	■	■	■	■	■
GAD-7		■	■	■	■	■	■	■
PHQ-9		■	■	■	■	■	■	■
ISI		■				■	■	■
CGI			■	■	■	■	■	■
TESS			■	■	■	■	■	■
SASE						■	■	■
SDSS		■	■	■	■	■	■	■
WHO-QOL-Brief		■	■	■	■	■	■	■
TAS-20		■				■	■	■
CERQ		■				■	■	■
LSAS		■	■	■	■	■	■	■
PDSS		■	■	■	■	■	■	■

*MINI6.0.0, Mini International Neuro-psychiatry Interview (Version 6.0.0); HAMA, Hamilton Anxiety Rating Scale; HAMD-17, the 17-item Hamilton Depression Scale; GAD-7, Generalized Anxiety Disorder-7; PHQ-9, Patient Health Questionnaire-9; ISI, Insomnia Severity Index; CGI, Clinical Global Impression*.

*TESS, Treatment Emergent Symptom Scale; SASE, Self-Assessment Scale of the Overall Efficacy and Satisfaction of Patients; SDSS, Social Disability Screening Schedule; WHO-QOL-Brief, World Health Organization Quality of Life Brief Form; TAS-20, Toronto Alexithymia Scale; CERQ, Cognitive Emotion Regulation Questionnaire; LSAS, Liebowitz social anxiety scale; PDSS, Panic Disorder Severity Scale*.

### Sample Size

We calculated the sample size based a significance level of 0.05 and with power of 0.8. Previous literature showed that the effective rate of single-drug combination psychotherapy (the reduction rate of HAMA after treatment is greater than or equal to 50% as effective treatment) was 70%, and the effective rate of single-drug therapy was 50%. We assumed that the medication plus AI-assisted psychotherapy group had a 10% higher response rate than the medication alone group. Assuming 20% as drop-out rate, the target enrollment is 354 patients in each group, and 708 in total (17).

### Discontinuation

Patients could withdraw or will be asked to withdraw from the study for the following reasons: (1) serious adverse events, (2) emergence of a new concurrent severe physical disease that is not well controlled, (3) exacerbation of any existing physical disease, (4) poor compliance, (5) any time when the patient asks to withdraw, and (6) certain situations in which the researchers consider it will be better for the patient to discontinue the treatment than to continue (e.g., increased suicide risk, participation in the study presents a significant burden to the patient, patient needs hospitalization). Patients will be clearly informed that they have the right to withdraw from the study at any time for any reason, during no matter screening or intervention period. The decision to withdraw a patient from the study will be made by the site principal investigator in consultation with the research team at the main research site.

## Measurements

Our primary efficacy measurement is the Hamilton Anxiety Scale (HAMA). Response is defined by a 50% reduction at least in total HAMA score. Secondary efficacy measurement is improvement in treatment at an early stage (score reduction in HAMA ≥25% at week 2 of treatment). Incidence of adverse events will be also recorded.

Other measurements include the Hamilton Depression Scale (HAMD-17), Clinical Global Impression (CGI), Treatment Emergent Symptom Scale (TESS), Social Disability Screening Schedule (SDSS), World Health Organization Quality of Life Brief Form (WHO-QOL-Brief), Generalized Anxiety Disorder-7 (GAD-7), Patient Health Questionnaire-9 (PHQ-9), Insomnia Severity Index (ISI), the Self-Assessment Scale of the Overall Efficacy and Satisfaction of Patients (SASE), Toronto Alexithymia Scale (TAS-20), Cognitive Emotion Regulation Questionnaire (CERQ), Liebowitz social anxiety scale (LSAS), Panic Disorder Severity Scale (PDSS). The HAMD-17, CGI, TESS, SDSS, PDSS will be administered by independent blind raters. Other scales will be self-assessed. Paradigm of negative pictures stimulation, facial expression emotion analysis, phonetic and semantic analysis are also included.

## Statistical Analysis

The aim of this trial is the explore whether medication plus AI-assisted psychotherapy has greater efficacy than medication alone in the treatment of anxiety disorders. The principal analyses will be conducted using the intention-to-treat sample. One-sided 5% significance level will be adopted, and corresponding 95% CIs will be calculated whenever possible. Continuous outcomes, the HAMA score reduction from baseline to week 2 and week 12, will be analyzed using analysis of variance and general linear mixed models. Treatment allocation, time, and interaction between treatment allocation and time will be the main predictors. Gender, age, education level, and baseline scores will be the covariates and treatment site will be a random effect in the model. The mixed models will be estimated using restricted maximum likelihood. The least square mean difference between the groups at 12 weeks will be tested as the main analysis. Other analyses will include comparisons at weeks 2, 4, 8, and 12, with adjustment for multiple testing. Multiple comparison issues of *post-hoc* analysis will be handled using Bonferroni adjustment. Dichotomous variables will be compared between groups using the chi-square test, Fisher's exact test, or generalized estimating equation. The above analyses will also be conducted with the follow-up data.

## Oversight

The project management team of Shanghai Mental Health Center will be responsible for the management of this trial. All the members of the team will meet once a week to discuss the progress of the project and issues requiring attention. The project manager will be responsible for organizing trainings of the researchers and raters at each trial sites. The trial statistician will be responsible for the establishment of data acquisition systems and in designing the clinical reporting forms.

Clinical research center of SMHC will guarantee the quality of the study. The clinical research center will be responsible for supervising the conducting of the study. There will be regular conferences every 3 months. The supervision might include patient recruitment, patient retention, protocol amendments, protocol violations, adverse events, data collection and recording, and timely reports of trial results. Additional meetings will be held when unforeseen circumstances occur. The clinical research center will also provide advice on all appropriate aspects of the trial if needed. We also hold meetings in smaller scales of the core researchers and supervisors every week, discussing the progress and problems during the conducting procedure.

## Discussion

Psychotherapy delivered in combination with medications produces better outcomes for patients with anxiety disorders than medication alone. However, psychological resources are very limited in China and the ability, to deliver psychotherapy to patients with anxiety disorders is therefore also limited. AI-assisted psychotherapy has the potential to address this problem and to be delivered in a standardized way in combination with medications. Our AI-based psychological chatbot app, XIAO AN, adopts multi-modal signal recognition and natural interaction technology, and combines virtual human technology to simulate a “real therapist.” It can deliver systematic psychological evaluation and treatment for anxiety disorder patients. At the same time, it can be trained to continuously optimize and upgrade by using AI deep learning technology. In this project, we will determine if in combination with medication it addresses anxiety disorders more effectively than medication alone.

## Trial Status

The trial is in the recruitment phase. The protocol version number is 2021-02 (January 13th, 2021). Recruitment began on May 6, 2021 and is expected to be completed by March 31, 2022.

## Ethics Statement

The studies involving human participants were reviewed and approved by Ethics Committee of Shanghai Mental Health Center. The patients/participants provided their written informed consent to participate in this study.

## Author Contributions

YX and JQ contributed to the conception of the study and obtained funding for the trial as the primary investigator. JQ, YWa, and SS collaboratively developed the study design and prepared the manuscript. SS did coordination work during the protocol implement. JQ, YWa, WZ, and RG developed the structure and content of XIAO AN with the supports of WJ, YWu, JT, YS, and JZ. SS and KL are the research coordinators. YWa translated the manuscript into English. ZZ gave suggestions in the language translation. YLu, XH, LaW, XW, YLi, QJ, and LiW are sub-PIs of the sub-centers. MZ, ZW, HL, and JH gave important suggestions about the study design. All authors read and approved the final manuscript.

## Funding

This study was funded by the Shanghai Hospital Development Center (Grant Number: SHDC2020CR1027B).

## Conflict of Interest

ZZ is employed by ChuanYu (Shanghai) Education Technology Co., Ltd. The remaining authors declare that the research was conducted in the absence of any commercial or financial relationships that could be construed as a potential conflict of interest.

## Publisher's Note

All claims expressed in this article are solely those of the authors and do not necessarily represent those of their affiliated organizations, or those of the publisher, the editors and the reviewers. Any product that may be evaluated in this article, or claim that may be made by its manufacturer, is not guaranteed or endorsed by the publisher.
